# Aperiodicity in Low Dimensions

**DOI:** 10.3390/ma19030446

**Published:** 2026-01-23

**Authors:** Pavel V. Avramov, Hao Tian, Li Li

**Affiliations:** 1 School of Physics, Harbin Institute of Technology, Harbin 150001, Chinalili.phys@hit.edu.cn (L.L.); 2 Department of Physics, Tomsk State University, 36 Lenin Ave., 634050 Tomsk, Russia

**Keywords:** low-dimensional solids, aperiodic crystals, quasicrystals, multiply twinned particles, nanodiamonds, decahedral symmetry, icosahedral symmetry, spin degeneracy, symmetry breaking

## Abstract

This review provides a comparative analysis of the structure and physical properties of low-dimensional aperiodic crystalline solids, aiming to elucidate the origin and nature of aperiodicity in reduced-dimensional lattices. The breakdown of periodicity in low-dimensional systems arises from several mechanisms, including the suppression of specific force constants, thermodynamic instabilities, and topological constraints associated with imperfect space filling. At the nanoscale, certain cubic crystalline materials can form finite, zero-dimensional multiply twinned particles (MTPs) with decahedral or icosahedral symmetry. These clusters lack translational invariance and experience intrinsic structural strain due to solid-angle mismatch at twin junctions, which limits their characteristic size and renders them finite aperiodic solids. Particular attention is devoted to the electronic and spin properties of pentagonally symmetric MTPs: icosahedral particles exhibit symmetry-protected spin degeneracy consistent with centrosymmetric lattices, whereas non-centrosymmetric decahedral particles may display spin polarization and emergent low-dimensional magnetism. Collectively, these systems illustrate the diverse physical origins, manifestations, and consequences of aperiodicity in low-dimensional crystalline matter.

## 1. Outline and Scope of the Review

This review provides a unified discussion of aperiodicity and quasiperiodic order in three-dimensional and low-dimensional crystalline systems, with particular emphasis on the geometric and topological origins of aperiodic structures. Section 2 introduces three-dimensional quasicrystals, beginning with a summary of their structural principles, symmetry properties, and higher-dimensional crystallographic description, followed by a discussion of their fundamental physical properties. Section 3 focuses on quasicrystallinity and aperiodicity in reduced dimensions, where geometric frustration and topological constraints play a central role. It addresses the departure from periodicity in free-standing, highly symmetric two-dimensional lattices, the topological instability of low-dimensional systems with multiple nonequivalent sublattices, and the role of intrinsic curvature, as formalized by Gauss’s Theorema Egregium and the Euler–Gauss–Bonnet theorem. This section further examines two-dimensional quasicrystals based on Penrose tilings, aperiodicity in incommensurate two-dimensional lattices, and finite-sized zero-dimensional aperiodic crystalline solids, including closed-shell multiply twinned sp^3^-bonded carbon and silicon clusters. Finally, Section 4 summarizes the key insights and highlights the common geometric and topological principles that govern the emergence of aperiodicity across different dimensionalities. Overall, the main focus of the review is to elucidate aperiodicity as an intrinsic consequence of symmetry, topology, and dimensional reduction rather than as an externally imposed structural motif.

## 2. Quasicrystals in Three Dimension

### 2.1. Basic Facts About Structure of 3D Quasicrystals

Historically, three-dimensional quasicrystals are well-studied aperiodic crystalline solids [1] discovered back in 1984. Quasicrystals are a distinct class of solid materials whose atomic arrangement exhibits long-range order and orientational symmetries forbidden in conventional crystals—e.g., 5-fold or icosahedral—yet they lack three-dimensional translational periodicity [2]. Unlike ordinary crystals, which repeat a unit cell in space, quasicrystals can be described mathematically using higher-dimensional geometry as projections of periodic structures from higher dimensions (such as 4D, 5D, or 6D space) onto 3D space [1,3,4], but they never exactly repeat in a periodic way. Penrose tiling [5,6], developed by mathematician Roger Penrose, is a two-dimensional analog that illustrates a nonrepeating but ordered pattern using a combination of shapes such as rhombi or kites.

Typical polymetallic quasicrystals are often complex intermetallic alloys (for example, Al–TM, with TM as a transition metal, or other multi-component combinations) and sometimes fall in the broader family of what are called Complex Metallic Alloys when their structural units form very large unit cells (effectively thousands of atoms) that approximate periodicity only over very large distances [7].

Because of this aperiodic yet ordered structure, quasicrystals can show rotational 5-fold, 10-fold, and 12-fold symmetries that are forbidden in periodic crystals under the classical Crystallographic Restriction Theorem [8,9]. Experimentally, such symmetries are observed via sharp Bragg peaks in diffraction—a hallmark of long-range order—but the diffraction pattern cannot be indexed with a small repeating unit cell [1].

Three-dimensional quasicrystals can be classified as follows:

1. Icosahedral quasicrystals, which display pentagonal and icosahedral symmetries, like Aluminum–Manganese alloys.
2. Decagonal quasicrystals with 10-fold symmetry in one direction and periodicity along another, like Aluminum–Nickel–Cobalt intermetallics.
3. Dodecagonal quasicrystals, which display 12-fold symmetry, are typically found in transition metal alloys. Example: Aluminum–Cobalt–Nickel systems.
4. Octagonal and other quasicrystals are rare quasicrystals that exhibit 8-fold or other non-crystallographic symmetries.

### 2.2. Fundamental Properties of 3D Quasicrystals

The unusual structural order of quasicrystals gives rise to a variety of physical properties that distinguish them from both conventional crystalline metals and amorphous alloys [7,10]. Many metallic quasicrystals often exhibit a pseudogap near the Fermi level, which strongly reduces the density of electronic states available for conduction. As a result, their electrical conductivity is much lower than typical metals—sometimes showing semiconducting or poor-metal behavior rather than good metallic conduction [7]. Quasicrystals are typically very hard, brittle, and resistant to deformation or wear. Their complex atomic arrangement reduces defect mobility, like dislocations that normally facilitate plastic deformation in metals, so they tend to fracture rather than deform plastically under stress [7]. Surface and chemical behavior: Many quasicrystalline alloys exhibit low surface energy, low friction, and good corrosion resistance. These features make them attractive for coatings, composite reinforcements, or surface-treatment applications, where wear resistance or non-stick behavior is desirable [7].

In addition, because their quasiperiodic order fundamentally deviates from periodic lattices, quasicrystals also show unconventional phonon properties and can host phason modes, which may influence thermal properties and lattice dynamics [11]. Polymetallic alloys and intermetallics, especially those involving Al with transition metals, or more complex mixtures are among the most common systems in which quasicrystals form [7]. As the number of atomic species and the size of structural units increase, lattice complexity grows, leading to emergent behaviors markedly different from simple alloys. Three-dimensional quasicrystals combine features of crystalline order like long-range order and the stability of atomic positions with non-periodic structure, which can suppress typical crystal defects and dislocation motion, giving rise to remarkable hardness and wear resistance [12].

Low thermal and electrical conductivity, along with good chemical stability and surface properties, make them promising for specialized applications, e.g., heat-insulating coatings, low-friction surfaces, composite reinforcements, or even photonic/optical uses in engineered quasicrystalline materials [7]. The overall stability depends strongly on composition, temperature, and processing history. Many polymetallic quasicrystals are metastable—formation often requires non-equilibrium processes like rapid cooling, melt spinning, and sputtering—though some can be stabilized as equilibrium phases by careful alloy design [12].

In three dimensions, aperiodicity manifests most prominently in quasicrystals and complex intermetallic phases, where long-range order without periodicity gives rise to unique combinations of mechanical, thermal, and electronic properties. Three-dimensional aperiodic structures exhibit low thermal conductivity, high hardness, enhanced resistance to wear and oxidation, and unconventional electronic responses, which have motivated their use in surface coatings, thermal barrier materials, and functional composites. More broadly, three-dimensional aperiodicity provides a framework for understanding and tailoring hierarchical order in complex solids, where structural complexity across multiple-length scales can be leveraged to stabilize unusual physical properties. Thus, while aperiodicity is not “utilized” in the conventional engineering sense, its intrinsic structural consequences are increasingly recognized as a resource for realizing novel functionalities in both low-dimensional and bulk systems.

In summary, three-dimensional quasicrystals exhibit a distinctive combination of metallic and nonmetallic properties. First, they display low thermal and electrical conductivity: despite being composed of metallic elements, quasicrystals conduct heat and electricity significantly less efficiently than conventional metals, often exhibiting behavior closer to that of semiconductors. Second, quasicrystals are characterized by high hardness and pronounced brittleness; they are highly resistant to plastic deformation yet difficult to machine or shape. Third, their unique surface structure results in low coefficients of friction and non-stick behavior, which has motivated their use in protective coatings and surface treatments. Fourth, many quasicrystalline alloys demonstrate excellent resistance to oxidation and corrosion. Finally, quasicrystals may exhibit unusual optical responses arising from their complex aperiodic order, including atypical light reflection and scattering, which makes them attractive for photonic and optoelectronic applications.

## 3. Quasicrystals in Low Dimensions

### 3.1. Departure from Periodicity of Free-Standing, Highly Symmetrical 2D Lattices

A fundamental source of aperiodicity in free-standing, low-dimensional crystalline membranes arises from their intrinsic thermodynamic instability. According to the the theorems of Landau–Peierls and Mermin–Wagner, long-range positional order in strictly 1D and 2D crystals is destabilized by thermal fluctuations, leading to a divergence of mean-square atomic displacements and preventing perfect long-range order [13,14]. When a 2D lattice is embedded in three-dimensional space, out-of-plane fluctuations additionally promote crumpling instabilities [15]. However, anharmonic coupling between bending and stretching modes suppresses these long-wavelength instabilities and stabilizes the membrane in a fluctuating, non-planar configuration [16,17].

Graphene provides the most prominent example of this behavior. As a strictly two-dimensional atomic crystal, it cannot remain perfectly flat at finite temperatures due to the thermal excitation of long-wavelength flexural modes, as implied by the Mermin–Wagner theorem [18,19]. Free-standing graphene therefore spontaneously forms out-of-plane deformations—intrinsic ripples—which minimize the membrane’s free energy by suppressing the divergence of thermal fluctuations and avoiding the elastic instability of an ideal flat sheet. These features emerge from the combined action of thermal flexural phonons and the sheet’s bending rigidity [20,21]. At elevated temperatures or in finite flakes, larger-amplitude, low-frequency, “paddle-like” deformations also appear, enhanced by edge effects, local strains, or defect-induced stresses; molecular dynamics simulations show that such modes fluctuate dynamically on picosecond–nanosecond timescales [22,23]. Out-of-plane fluctuations in graphene are strongly coupled with in-plane stretching modes, an anharmonic interaction that renormalizes the effective bending rigidity and ensures thermodynamic stability while still permitting significant height undulations [21].

These theoretical predictions are supported by direct experimental observations: high-resolution TEM and STM studies reveal intrinsic ripples in suspended graphene, with amplitudes of ~0.5–1 nm and wavelengths of 5–25 nm, fully consistent with simulations [22,23,24,25,26]. Similar behavior is expected for other nominally planar, highly symmetric 2D crystals—including h-BN [27], graphane [28], graphdiyne and its derivatives [29,30], γ-graphyne [31], holey graphyne [32], g-C_3_N_4_ [33], g-C_4_N_3_ [34], and diamanes [35,36]—which, according to the same theoretical arguments, are thermodynamically unstable in perfectly flat configurations. The stability issues of 2D flat silicene and germanene were discussed as well [37]. Their spontaneous nanometer-scale corrugations represent self-generated mechanisms for redistributing strain and suppressing long-wavelength instabilities. Thus, intrinsic rippling in free-standing 2D materials exemplifies a general thermodynamic pathway to aperiodicity in highly symmetric, low-dimensional crystalline lattices.

### 3.2. Topological Instability of Low-Dimensional Lattices with Multiple Nonequivalent Sublattices: Topology Conservation, Theorem, Theorema Egregium, and Euler–Gauss–Bonnet Theorem

Slight, systematic distortions of the structural units can themselves destroy long-range crystalline order in hypothetical low-dimensional solids by reducing or breaking the ideal lattice symmetry [38,39,40,41,42,43]. It is important to note that, in low-dimensional lattices, one (in 2D) or two (in 1D) of the effective elastic force constants are intrinsically zero, removing the corresponding restoring forces for long-wavelength modes and thereby amplifying fluctuations that ultimately prevent the preservation of true long-range crystalline order.

**Figure 1:**
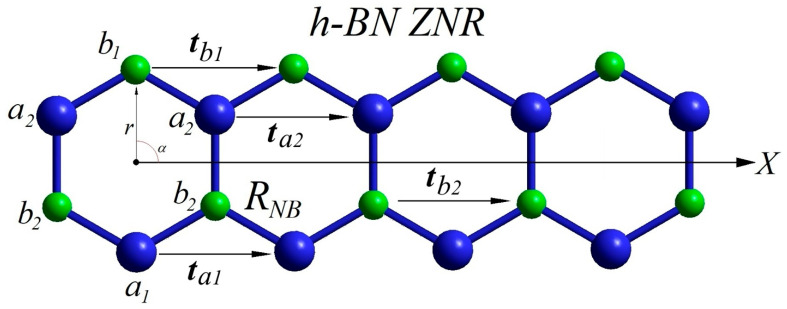
A fragment of 1D *h*-BN zigzag narrow nanoribbon of one B_3_N_3_ hexagonal fragment width, oriented along the *X*-axis. *a*_1_, *a*_2_, *b*_1_, and *b*_2_ are nonequivalent sublattices filled by N (*a*_1_ and *a*_2_, in blue) and B (*b*_1_ and *b*_2_, in green) atoms, respectively. *R_NB_* is the length of interatomic bonds; $t_{a1}$, $t_{a2}$, $t_{b1}$, and $t_{b2}$ are translation vectors associated with *a*_1_, *a*_2_, *b*_1_, *b*_2_ sublattices, respectively. *α* is the angle between *X* direction and *r*, where *r* is an atomic radius vector. Adopted from [39].

A convenient elementary example is a one-dimensional zigzag *h*-BN nanoribbon (*h*-BN ZNR) of minimal width—one B_3_N_3_ hexagonal fragment (Figure 1). Such a narrow ribbon already contains four symmetry-inequivalent sublattices, meaning the translationally repeated unit is not a single identical atom but a small basis with nonequivalent sites.

Without loss of generality, let us label the two nonequivalent nitrogen sublattices as *a*_1_ and *a*_2_ and the two nonequivalent boron sublattices as *b*_1_ and *b*_2_. These are associated with four symmetry-distinct translation vectors $t_{a1},t_{a2},t_{b1},\;and\;t_{b2}$ directed along the ribbon axis *X*. The local environments differ: the atomic rows along the outermost edge have coordination number *Z* = 2, while the inner atoms have coordination *Z* = 3. In the notation introduced above, one finds, for example, that an *a*_1_ (N) atom is bonded to two *b*_2_ (B) atoms, whereas a *b*_1_ (B) atom is bonded to two *a*_2_ (N) atoms. Likewise, the inner sublattices have mixed neighborhoods: an *a*_2_ (N) atom has two *b*_1_ and one *b*_2_ boron neighbors, while a *b*_2_ (B) atom has two *a*_1_ and one *a*_2_ nitrogen neighbors.

For simplicity, let us assume a single characteristic B–N bond length *R_NB_* for all nearest-neighbor bonds; nevertheless, because the basis atoms, boundary conditions, coordination numbers, and local environments of the four sublattices are inequivalent, their on-site (local) structural parameters will generally differ. One can write the following:

$$a_{a1}\neq a_{a2}{\neq a}_{b1}\neq a_{b2}$$

where $a_{ai}$ and $a_{bj}$ denote representative local structural parameters (for instance, local bond-angle averages, local lattice offsets, or on-site potentials) characterizing sublattices *a_i_* and *b_j_*. These inequivalences—in combination with reduced dimensionality—provide a simple microscopic route for the spontaneous breakdown of perfect translational symmetry and the emergence of aperiodic or modulated ground states in low-dimensional materials.

The *a*_1_ and *b*_1_ sublattices (*Z* = 2) possess different force constants, *Q*_1_ and *Q*_2_, along *X* caused by symmetry and environment differences in edge N, and B atoms, even *a*_2_-*b*_1_-*a*_2_ and *b*_2_-*a*_1_-*b*_2_ angles, and the *R_NB_* length of all N-B bonds have the same values as perfect 2D *h*-BN. The force constant *Q* along *X* and *R_NB_* for *a*_2_ and *b*_2_ sublattices (*Z* = 3) keep the initial value of perfect 2D *h*-BN. For a perfect hexagonal lattice, $\left|t_{a1}\right|=\left|t_{b1}\right|=\sqrt{3}R_{NB}$. So, for *t*_a2_ and *t_b_*_2_ and the force constants, one can write

$$Q_{1}\neq Q_{2}\neq Q$$

$$a_{a2}=a_{b2}$$

Taking into account symmetry restrictions, for the stress energies *E_i_* and forces acting on *a_i_* and *b_i_*:

$$E_{a2}=E_{b2}$$

$$E_{a1}=E_{b1}$$

$$F_{a2}=F_{b2}$$

$$F_{a1}\neq F_{b1}$$

Symmetrical non-equivalency of the forces acting on boundary B and N atoms ($F_{a1}\neq F_{b1})$ creates mechanical stress and structural curvatures of ultranarrow *h*-BN ZNR and other similar zigzag heteroatomic nanoribbons [43].

For 2D, symmetrically perfect hexagonal lattices, the torques $\tau_{i}$ caused by uncompensated forces acting on nonequivalent sublattices are

τa2=RNB·Fa2·sin16π=RNBFa22

τb2=RNB·Fb2·sin−16π=−RNBFa22

$$\tau_{b2}=-\tau_{a2}$$

and *a*_2_ and *b*_2_ torques symmetrically compensate each other.

For the *a*_1_ and *b*_1_ sublattices of zigzag nanoribbons, *α* angles are equal to −π2 and π2, respectively, and

$$\left|r_{a1}\right|=\left|r_{b1}\right|=R_{NB},$$

so,

τa1=RNB·Fa1·sin−π2=−RNBFa1

τb1=RNB·Fb1·sinπ2=RNBFb1

with $\tau_{b1}$ and $\tau_{a1}$ oriented in opposite directions. The total torque acting on the nanostructure is the sum of the torques. Since $F_{a1}\neq F_{b1}$

$$\tau_{a1}+\tau_{b1}=R_{NB}\left|F_{b1}\right|-R_{NB}\left|F_{a1}\right|=R_{NB}\left(\left|F_{b1}\right|-\left|F_{a1}\right|\right)\neq 0$$

and the torques do not compensate each other. Since both nonequivalent torques associated with *a*_1_ and *b*_1_ sublattices are perpendicular to the plane of the *h*-BN ZNR lattice and oriented in opposite directions, they lead to displacements of the atoms with the formation of a cone fragment and departure from linear translation symmetry [42,43].

In particular, this conclusion was convincingly confirmed by DFT electronic structure calculations [43]. In the study, the atomic and electronic structures of various narrow zigzag nanoribbons with finite lengths were analyzed. It was found that edge asymmetry induces a uniform curvature of the ribbons due to structural stress within the aromatic ring plane. Narrow graphene nanoribbons terminated with fluorine on one side exhibit a pronounced out-of-plane bending, indicating that the nanoribbon locally approximates a fraction of a conical surface. This intrinsic curvature disrupts the perfect periodicity of the lattice and leads to the systematic cancelation of the dipole moment and formation of 1D aperiodic solid. Both in-plane and out-of-plane curvatures of these thin arcs facilitate their closure into nanorings or cone-like fragments, with very large diameters keeping a short-order crystalline nature. For instance, a planar giant arc and a closed ring of *h*-BN with a diameter of 105 nm were successfully modeled, illustrating this curvature-induced structural adaptation.

Gauss’s *Theorema Egregium* [44,45,46] establishes that the *local* Gaussian curvature K of a surface is an intrinsic geometric quantity, fully determined by the surface metric and independent of its embedding in three-dimensional space. Consequently, the curvature can be detected by measurements performed entirely within the surface and is invariant under bending without stretching. This result is fundamental for polygonal and lattice-based surfaces, as it implies that curvature introduced by topological defects must be encoded in intrinsic angular relations rather than in extrinsic shape alone.

For a discrete surface composed of perfectly planar, rigid polygons (with torsion angles equal to zero), the intrinsic metric is fully specified by edge lengths and interior angles. The discrete analog of the Gaussian curvature therefore appears as an angle defect concentrated at the vertices. For a polygonal (piecewise flat) surface, the Gaussian curvature at vertex v is defined as

$$K_{v}=2\pi -\sum_{i}\theta_{v,i},$$

where $\theta_{v,i}$ are the interior angles of all polygonal incidents at that vertex. This expression is the direct discrete counterpart of smooth Gaussian curvature and follows from Gauss’s theorem applied locally.

Perfect hexagonal tiling provides [47] a flat reference state: three hexagons meet at each vertex, each contributing an interior angle of 120°, yielding

$$K_{v}=2\pi -3\times 120{}^{\circ}=0.$$

Thus, hexagons carry no intrinsic Gaussian curvature in two-dimensional hexagonal lattices.

Replacing a hexagon with a pentagon or a heptagon introduces a wedge disclination into the lattice. For a regular pentagon and heptagon, the interior angles are 108° and approximately 128.57°, respectively. The resulting discrete Gaussian curvatures per affected vertex are

$$K_{v}^{p}=360{}^{\circ}-(120{}^{\circ}+120{}^{\circ}+108{}^{\circ})=12{}^{\circ},$$

$$K_{v}^{h}=360{}^{\circ}-(120{}^{\circ}+120{}^{\circ}+128.57{}^{\circ})\approx -8.57{}^{\circ}.$$

Although the angle defect is computed from planar interior angles, it represents the holonomy of parallel transport and therefore corresponds to the integrated Gaussian curvature, which is naturally interpreted as a solid angle. The numerical use of degrees is conventional; after normalization by 2π, the result is dimensionless and equivalent to a solid angle measured in steradians.

A single pentagon has five vertices; therefore, its total intrinsic Gaussian curvature is

$$K_{pent}=5\times 12{}^{\circ}=60{}^{\circ}=\frac{\pi }{3}\;sr$$

Analogously, a heptagon contributes a total negative Gaussian curvature of the same magnitude,

$$K_{hept}\approx 7\times \left(-8.57{}^{\circ}\right)\approx -60{}^{\circ}=-\frac{\pi }{3}\;sr$$

The global constraint on the curvature is provided by the *Euler–Gauss–Bonnet theorem* [44,45,48,49]. For a compact, oriented, two-dimensional Riemannian manifold M without a boundary, the total Gaussian curvature satisfies

$$\int_{M}K\;dA=2\pi \;\chi (M),$$

where χ(M) is the Euler characteristic. For a sphere, χ=2, and thus, the total curvature equals 4π.

For a closed polygonal surface, the theorem takes the discrete form

$$\sum_{v}\left(2\pi -\sum_{i}\theta_{v,i}\right)=2\pi \;\chi$$

where the Euler characteristic is given by χ=V−E+F, with V, E, and F denoting the numbers of vertices, edges, and faces, respectively. This result is exact and does not rely on smoothness assumptions.

Accordingly, any closed polygonal shell—including those composed exclusively of pentagons and hexagons [50]—must satisfy

$$\sum_{v}K_{v}=4\pi \;sr.$$

Since each pentagon supplies a curvature of π/3, exactly twelve pentagons are required,

$$12\times \frac{\pi }{3}=4\pi \;sr.$$

forming a closed-shell polygonal lattice consisting solely of hexagons and pentagons.

Finally, it is important to note that the frequently quoted value of 30° per pentagon does not represent the Gaussian curvature. Instead, it arises from a geometric projection of the intrinsic (solid-angle) curvature into a two-dimensional plane cross section and therefore corresponds to an extrinsic plane-angle measure rather than to an intrinsic Gaussian curvature.

**Figure 2:**
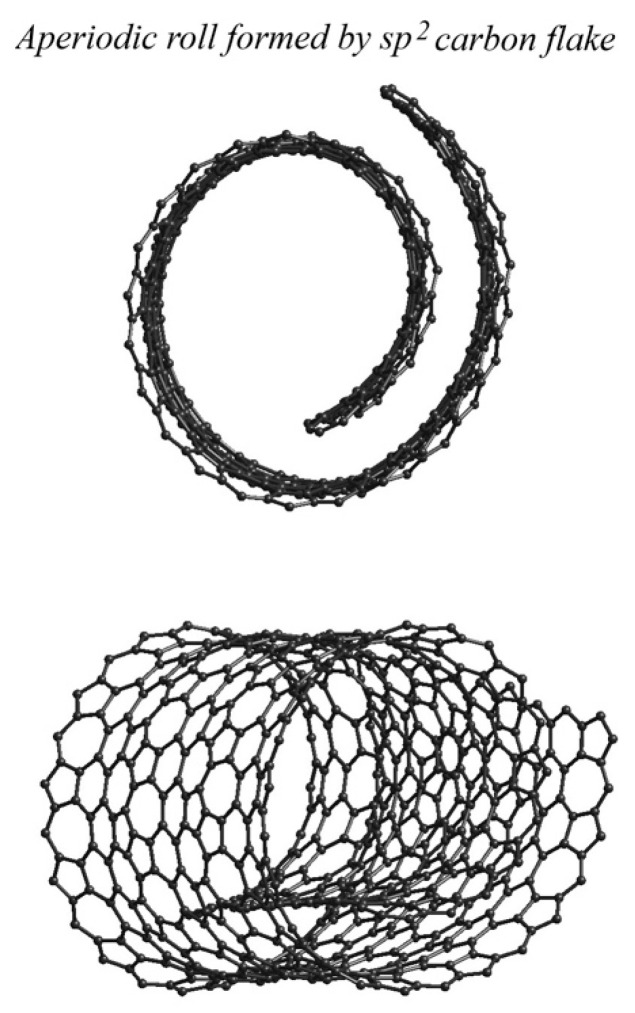
Structure of one low-dimensional aperiodic crystalline lattice formed by regular combination of pentagonal, hexagonal, and heptagonal sp^2^-hybridized carbon rings. Structural stress forms distinctive aperiodic bent roll [39].

Multiple extended 1D and 2D lattices containing several nonequivalent sublattices—constructed from combinations of one-atom-thick pentagonal, hexagonal, heptagonal, and octagonal aromatic and antiaromatic carbon rings and fragments—have been investigated [38,39] using a combination of experimental approaches. In such systems, translational symmetry and long-range crystalline order are inherently disrupted. As a result, these aperiodic lattices can adopt a variety of curved or irregular morphologies, including bent rolls, screw-like structures, irregular flakes, atomistic clusters, and other non-periodic arrangements (Figure 2).

For a rigorous description of the phenomena leading to the disruption of periodicity and formation of aperiodic low-dimensional crystalline solids, the Topology Conservation Theorem was introduced, formulated, and proven [38,39]. The Theorem states the following:

*“To conserve planar topology of one-unit-cell-thick planar crystals with negligible stabilizing force constant in the perpendicular direction, and to avoid uncompensated mechanical stress perpendicular to the regular lattice plane, the free-standing constituting fragments (unit cells) must perfectly fit the low-dimensional space. Due to the leading contribution of the stretching force constants to total energy, any small regular structural mismatch should accumulate and lead to motion of the crystalline lattice in the perpendicular direction to the plane to compensate the accumulated mechanical stress.”*

Only a few one-atom-thick 2D lattices satisfy the mandatory requirements of the theorem, since their structural fragments perfectly fill the planar 2D space and satisfy symmetry restrictions. These include the planar hexagonal and triangular lattices of graphene, *h*-BN, graphane, graphdiynes, and γ- and holey-graphynes, g-C_3_N_4_, and g-C_4_N_3_.

Two corollaries of the Topology Conservation Theorem can also be formulated. First, any form of external pressure—positive or negative—can effectively stabilize planar 2D lattices that are prone to deformation by alleviating the internal structural stress. Second, in stochastic atomistic lattices formed by structural units that do not perfectly fill the low-dimensional space, structural stress and the resulting regular deformations cannot accumulate due to the mutual compensation of displacements in opposite directions.

The validity of the first corollary is illustrated by the experimental fabrication of InGaAs/GaAs micro- and nanotubes with controllable inner diameters ranging from 4 μm to 4 nm, achieved by the selective etching of a stabilizing sacrificial AlAs substrate [51]. In this system, the GaAs/InAs pair exhibits a 7.2% lattice mismatch. Upon removal of the stabilizing AlAs substrate, a (2 ML)GaAs/(2 ML)InAs vertical heterostructure spontaneously rolls into a nanotube, with the GaAs fragment forming the inner layer. Here, the AlAs substrate acts as an effective negative pressure, maintaining the perfectly planar topology of external GaAs/InAs heterostructure during the initial stages of synthesis.

The validity of the second corollary is demonstrated by the experimental synthesis of a one-atom-thick sp^2^-hybridized carbon membrane with a random arrangement of four-, five-, six-, and seven-membered carbon rings, produced via electron irradiation of graphene [52,53]. The random distribution of defects eliminates the periodicity of the parent sp^2^ graphene lattice and prevents the accumulation of mechanical stress associated with the introduction of various types of defects.

It is important to note that both Gauss’s Theorema Egregium [44,45,46] and the Euler–Gauss–Bonnet theorem [44,45,48,49] are derived under the assumption of perfectly planar and rigid structural units—equivalently, infinitely large force constants are associated with out-of-plane bending and torsional deformations. In reality, this assumption does not hold for lattices that are only one-unit-cell thick. In such systems, the corresponding force constants are finite (albeit small), which permits the bending and torsion of the structural units and leads to complex three-dimensional deformations of structural units in particular and whole nanolattices in general [39,54]. As a result, predicting the atomic structure of low-dimensional materials becomes substantially more challenging, since simple analytical and purely geometric methods cannot be directly applied to the analysis and prediction of their equilibrium configurations.

### 3.3. Two-Dimensional Quasicrystals Based on Penrose Tilings

Two-dimensional quasicrystals constitute a distinct class of atomistic lattices that exhibit long-range orientational order in the absence of translational symmetry. Among the most influential mathematical frameworks for describing such structures are the Penrose tilings introduced by R. Penrose in the 1970s [5,6]. These tilings demonstrated that a strictly aperiodic yet perfectly ordered 2D space filling can be generated using only a small set of prototiles—originally the “star,” “boat,” and “diamond” shapes of the P1 set [55]. Subsequent refinements reduced this set to the P2 “kite-and-dart” tiling and later to the P3 tiling composed of two rhombi of equal side lengths but different interior angles. The accompanying matching rules ensure a nonrepeating pattern with local 5-fold rotational symmetry, a feature prohibited in periodic crystals but characteristic of many experimentally observed quasicrystalline phases. These mathematical constructions now serve as a cornerstone for modeling atomic arrangements in 2D quasicrystalline alloys and artificial nanostructures.

**Figure 3:**
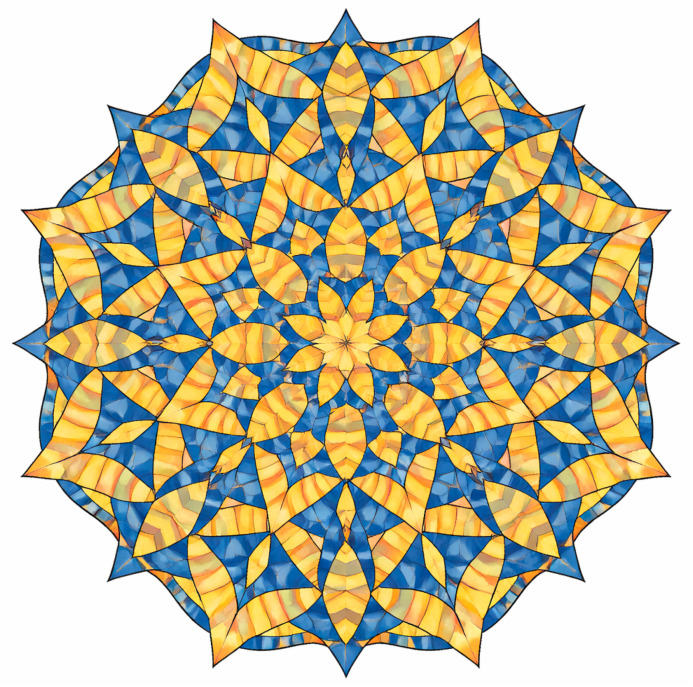
Penrose tiling with global decagonal symmetry constructed from kite and dart prototiles. All tile edges are of equal length, while interior angles are multiples of 36°, enforcing quasiperiodic order via golden-ratio-based matching rules. Color coding distinguishes the two prototiles and highlights the formation of characteristic 5-fold star and rosette motifs. The absence of translational periodicity, together with the presence of long-range orientational order and inflation symmetry, illustrates the essential geometric features of two-dimensional quasicrystals.

Penrose tilings represent a canonical example of two-dimensional quasiperiodic order, characterized by long-range orientational symmetry in the absence of translational periodicity. Let us consider an example of the tiling (Figure 3) constructed from two elementary prototiles—the kite and the dart—whose geometry is governed by angles that are integer multiples of 36° and by length ratios fixed by the golden ratio $\varphi =\left(1+\sqrt{5}\right)/2$ [5,6]. Although each tile has equal edge lengths, strict matching rules enforce a non-periodic arrangement that precludes crystallographic symmetry while preserving global 5-fold (decagonal) rotational order. The resulting structure exhibits hierarchical self-similarity through inflation–deflation symmetry, with recurrent star- and rosette-like motifs appearing at multiple lengths of scales. Intrinsically, the tiling is flat, yet it encodes topological frustration analogous to that found in quasicrystalline atomic arrangements, making it a paradigmatic geometric model for understanding quasiperiodicity, forbidden symmetries, and electronic or phononic states in aperiodic solids.

Quasiperiodic Penrose tilings provide a natural geometric framework for understanding electronic degeneracy in systems that lack translational symmetry yet preserve long-range orientational order. The local 5-fold and decagonal motifs inherent to the tiling impose non-crystallographic point symmetries on the electronic Hamiltonian, leading to degeneracies that are protected not by Bloch periodicity but by rotational symmetry and time-reversal invariance. In particular, star- and rosette-like clusters act as symmetry centers, where electronic states transform according to either irreducible representations of the dihedral groups *D*_5_ or *D*_10_, giving rise to quasi-degenerate energy levels analogous to those observed in atomic clusters and quasicrystalline approximants. The hierarchical inflation symmetry further introduces multiscale organization of the electronic spectrum, promoting critical states with power-law localization rather than fully extended Bloch waves or exponentially localized states. As a consequence, electronic degeneracy in Penrose-tiling-based systems emerges as an intrinsically geometric effect, rooted in quasiperiodic symmetry and protected by time-reversal symmetry, rather than as a consequence of lattice periodicity or band folding, fundamentally distinguishing quasicrystals from both periodic crystals and disordered solids.

The aperiodicity inherent to Penrose-type lattices gives rise to a rich spectrum of physical phenomena not accessible in periodic materials. Electronic states in Penrose-based quasicrystals often display critical or multifractal behavior, occupying an intermediate regime between fully delocalized Bloch waves and exponentially localized states, with profound implications for quantum transport [1,2]. Their vibrational spectra similarly deviate from those of periodic crystals: well-defined phonon branches are replaced by dense, highly fragmented vibrational continua, leading to unusual thermal conductivity and mechanical responses. These properties have motivated extensive theoretical studies and experimental emulation in metallic thin films, photonic lattices, and phononic metamaterials, where Penrose symmetry can be imposed with nanometer-scale precision [3,4,7]. In photonic and plasmonic realizations, for example, the absence of translational symmetry results in broadband photonic pseudogaps and unconventional wave localization patterns, further highlighting the versatility of aperiodic order.

Experimentally, genuine 2D quasicrystals with Penrose-type order have been observed in several metallic alloy systems, notably Al–Mn–Si and related ternary phases, in which high-resolution diffraction measurements reveal the characteristic 5-fold symmetry and dense Fourier spectra predicted by Penrose tilings [3]. More recently, advances in atomically precise fabrications—such as molecular self-assembly, controlled surface adsorption, and nanolithography—have enabled the creation of artificial 2D quasicrystalline monolayers and metasurfaces with tunable structural parameters [6]. Single-layer quasicrystalline films have also been obtained through liquid-phase exfoliation and related methods [56,57]. At the nanoscale, however, these aperiodic lattices naturally develop local distortions including strain, dislocations, and crack-like defects, reflecting the constraints imposed by finite sample sizes and boundary effects. Theoretical modeling has played a crucial role in predicting and interpreting these features, typically achieving strong consistency with experimental data [56,57]. Importantly, the irregular local environments characteristic of aperiodic tilings give rise to highly reactive edge and surface sites, which significantly enhance catalytic activity [58,59,60,61] and promote complex surface chemical processes [62]. Together, these observations underscore the unique interplay between mathematical aperiodicity and physical functionality in 2D quasicrystals.

### 3.4. Aperiodicity in 2D Incommensurate Lattices

Despite the rapid development of quasicrystal science over the past four decades, the literature specifically devoted to two-dimensional incommensurate (IC) quasicrystals remains relatively scarce. This is unsurprising, since most experimentally characterized aperiodic materials belong either to classical polygonal 2D QC families or to 3D icosahedral quasicrystals, while IC systems require more subtle structural analysis and advanced diffraction interpretation. Two broad categories of quasicrystalline lattices are commonly distinguished: polygonal (2D) QCs and icosahedral (3D) QCs, each exhibiting characteristic non-crystallographic symmetries and lacking translational periodicity [63]. Polygonal 2D QCs can display local rotational symmetries of 5-, 8-, 10-, or 12-fold, whereas icosahedral QCs exhibit fully three-dimensional aperiodicity.

A defining feature of crystalline matter is the presence of long-range order (LRO), which manifests as discrete Bragg peaks in X-ray and electron diffraction due to the translational invariance (TI) of the underlying atomic arrangement [64]. Amorphous solids, by contrast, exhibit only short-range order (SRO) and lack Bragg reflections, displaying broad halos instead [65]. Between these extremes lie three major classes of non-TI yet ordered materials: quasicrystals (QCs), incommensurate modulated crystals (IMCs), and incommensurate composite crystals (ICCs) [66,67]. All three families maintain long-range orientational order combined with characteristic diffraction patterns yet break translational symmetry. Their structural description requires higher-dimensional crystallographic approaches, where reciprocal space vectors span a superspace of dimensionality D ≥ d, with equality holding only for periodic crystals [68]. In this framework, additional reciprocal-space dimensions represent modulation waves or aperiodic displacements.

Incommensurate modulated crystals arise when an otherwise periodic lattice is modulated by one or more waves whose wavelengths are incommensurate with the underlying unit cell. For a single-q modulation, the displacement field can be written schematically as follows:

$$f\left(x\right)=A_{1}\sin\left(2\pi k_{1}x\right)+A_{2}\sin\left(2\pi k_{2}x\right)$$

where the ratio c=k1k2 determines the nature of the system. If c is rational, the modulation is periodic, and the solid can be described using an enlarged unit cell. If c is irrational, the modulation cannot be accommodated by any finite supercell, resulting in an aperiodic IC structure.

For irrational c, the Fourier expansion of $f\left(x\right)$ separates into commensurate and incommensurate components, producing a quasi-dense set of diffraction peaks. These reflections may be indexed as integer combinations of superspace basis vectors, generating a reciprocal lattice of dimensionality D > d [68]. Increasing the number of modulation vectors enriches the geometry of the corresponding atomic surfaces in superspace and modifies the allowed phonon and phason excitations. As a result, IMCs exhibit pseudo-Brillouin zones (PBZs) around the strongest Bragg peaks and support unconventional mixed phonon–phason dispersion relations, which strongly affect transport, elasticity, and thermal properties.

IMCs represent only one branch of the broader class of incommensurate materials. Incommensurate composite crystals, consisting of two interpenetrating periodic and aperiodic sublattices, form another important category, but such systems fall outside the scope of the present discussion.

A paradigmatic example of a 2D incommensurate aperiodic lattice is 30° twisted bilayer graphene (TBlG) [69,70,71]. Rotating one graphene sheet relative to the other by exactly 30° eliminates any possibility of forming a periodic moiré superlattice. Instead, the system becomes strictly incommensurate, forming a dodecagonal quasicrystalline arrangement of overlapping hexagonal lattices [72].

The 30° twist dramatically enhances interlayer coupling by aligning the Dirac cones of the two layers in a mirrored configuration. This contrasts sharply with small-angle twisted bilayers, where electronic bands can be described using periodic supercells [72]. Angle-resolved photoemission spectroscopy (ARPES) experiments reveal multiple Dirac cones arranged with 12-fold rotational symmetry, confirming the emergence of a quasicrystalline electronic structure [71]. The unusual band topology in TBlG enables the direct exploration of non-TI Dirac electron states and even supports higher-dimensional topological phenomena, such as the 4D quantum Hall effect projected into the 2D QC lattice [73].

The study of TBlG has revitalized interest in 2D incommensurate systems because it provides a clean, experimentally accessible platform where the electronic, vibrational, and optical properties of aperiodic lattices can be probed with unprecedented precision. The absence of periodicity leads to fragmented miniband structures, the suppression of Umklapp scattering, and unconventional localization behavior that cannot arise in periodic 2D materials.

Following the second corollary of the Topology Conservation Theorem (see above [38,39]), the loss of translational periodicity in quasicrystals and incommensurate lattices suppresses the accumulation of coherent elastic distortions. In periodic low-dimensional materials, structural mismatch accumulates coherently, often driving the lattice to buckle, ripple, or undergo symmetry-lowering distortions. In contrast, aperiodicity enforces stochastic local environments, causing mechanical stresses to cancel rather than accumulate. As a result, incommensurate 2D lattices and quasicrystals remain topologically stable despite local strain caused by the mismatch of structural local fragments.

This insight explains why incommensurate graphene bilayers, Penrose-type 2D quasicrystals, and various IC modulated structures maintain structural integrity even when composed of fragments that individually possess mismatched symmetry or bonding preferences. The absence of long-range translational registry prohibits the buildup of elastic frustration and thus stabilizes otherwise incompatible atomic arrangements.

### 3.5. Zero-Dimensional Finite-Sized Aperiodic Crystalline Solids Based on Closed-Shell Multiply Twinned sp^3^ Carbon and Silicon Clusters

Closed-shell multiply twinned nanodiamonds (see, for example, [74,75,76,77]) can be regarded as zero-dimensional aperiodic crystalline solids, in which well-defined local crystallinity coexists with the absence of a long-range translational order. Multiple twinning refers to the incorporation of several twinned regions within a single crystal, a feature that significantly influences the particle’s structural stability, electronic properties, and response to external stimuli.

The first reported closed-shell, star-shaped diamond, discovered in South Africa in 1932, weighed an enormous (see below) 1.8 carats and measured 8 mm [78], highlighting the morphological importance of {111} facets and their role in structural stability. Pentagonal symmetry diamonds, including decahedral, star-shaped decahedral, and icosahedral ones, have been observed both in nature and synthetically [79,80,81,82]. The synthetic formation of such structures has been achieved via chemical vapor deposition in carbon plasma containing CH_4_/H_2_ mixtures at ~50 Torr and 650 °C on (100) or (111) surfaces of monocrystalline silicon [83,84,85]. Their formation arises from twinning processes during nucleation on (111) surfaces, associated with packing defects and the reorientation of crystallographic axes [77,79,81,82,86,87,88]. Specifically, two packing defects lead to icosahedral structures, while three defects result in icosahedral particles [83,89].

Icosahedral diamond particles have been experimentally observed over a wide range of effective sizes, from tens of nanometers to several micrometers, often exhibiting characteristic 5-fold twinning and, in many cases, distinctive concave dimples at the vertices. Early CVD studies reported icosahedral twins ranging from 0.5 to 5 μm without noticeable vertex dimpling [77], whereas subsequent work identified ~1 μm particles with pronounced concave vertices [79], ~5 μm particles with well-defined dimples [80], 50 nm icosahedra lacking vertex dimples [81], 0.4–1 μm twins exhibiting strong vertex concavity [83], and ~2 μm particles with distinct dimples on all vertices [90].

**Figure 4:**
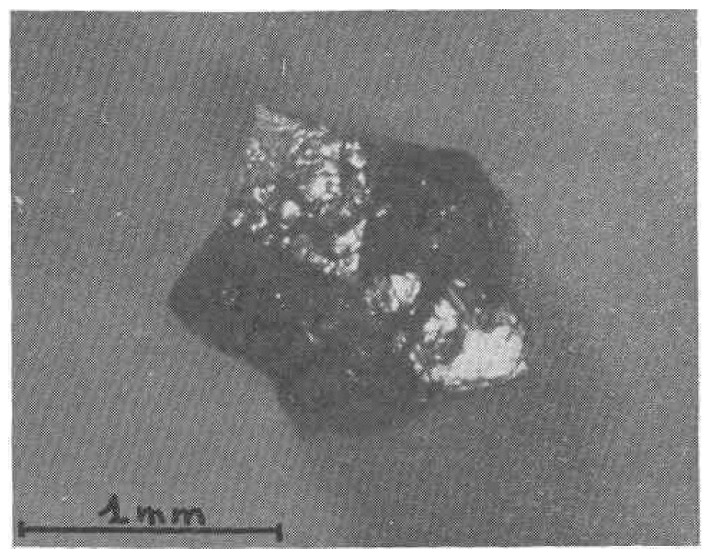
Cyclic (“Repeated” in original publication) twin diamond from Tortiya Mine, Ivory Coast [70]. Adapted from Figure 1, Ref. [76]. Reproduced with permission from Ref. [76]. Copyright [1972] [Mineralogical Society of America].

Also, it is worth noting that few other publications are devoted to the theoretical and experimental investigation of pentagonal and icosahedral nanodiamond structures, which are directly relevant to aperiodic, multiply twinned carbon lattices [91,92]. In particular, although not focused exclusively on diamonds, Lee and Glotzer [91] demonstrated through molecular simulations the formation and stabilization of 5-fold and icosahedral twinned clusters in hard-particle systems, highlighting the fundamental role of entropy and twin-boundary energetics in the emergence of multiply twinned structures with 5-fold and icosahedral symmetries. Experimentally, features characteristic of icosahedral diamond morphologies—such as pentagonal dimples at 5-fold junctions of (111) facets—have been observed in hot-filament chemical vapor deposition diamond crystals [92], suggesting non-classical growth pathways that favor multiply twinned and 5-fold-symmetric motifs. Taken together, these recent studies, combining computational modeling and crystallographic observations, underscore the continuing relevance of pentagonal and icosahedral structural motifs for understanding the formation, stability, and physical properties of non-periodic carbon nanostructures. The image of the historical pentagonal symmetry diamond [93] is presented in Figure 4.

The first recognized icosahedral diamond twins were described by Matsumoto and Matsui in 1983 [77], who demonstrated that these finite-sized CVD-grown particles are composed of twenty tetrahedral *fcc* diamond fragments joined along (111) planes via single-layer hexagonal interfaces. Electron diffraction and SEM analyses confirmed perfect 20-fold cyclic twinning and revealed that small gaps may form along some twin boundaries due to {100} truncations of (111)-bound fragments, although many particles are gap-free. These highly symmetric particles thus represent true finite-sized, radially ordered 3D crystalline solids without translational invariance, fundamentally distinct from classical 3D quasicrystals.

The formation of 20-fold icosahedral twins has been attributed to three stacking errors occurring during growth [94,95]. Kinetic Monte Carlo simulations show that two stacking faults produce decahedral forms, whereas three faults generate perfect icosahedral multiply twinned particles [83,94]. Using hot-filament CVD, high-quality micrometer-scale icosahedral diamonds with effective dimensions up to ~4 μm have been synthesized [96], and atomistic modeling suggests that the C_20_H_20_ dodecahedrane molecule can serve as a structural embryo for these icosahedral diamond MTPs.

Experimental studies conducted over several decades demonstrate that decahedral and star-shaped decahedral diamond particles appear across a remarkably wide size range—from hundreds of nanometers to several hundreds of micrometers—and frequently exhibit characteristic vertex dimples arising from 5-fold cyclic twinning. Systematic observations include the following: ~0.5 μm 5-fold particles without dimples [77]; ~0.5 μm particles with dimples and a localized twin-boundary misfit [77]; ~1 μm pentagonal stars with distinct concave vertices [79]; 50 nm decahedral twins [81]; star-shaped 5-fold particles of ~1 mm [76]; star-decahedral particles with dimples of ~1 μm and 1.5–15 μm [83]; dimpled decahedra of unspecified dimensions [82]; and a broad distribution of dimpled decahedra from 300 nm to 4 μm [96]. The earliest known example—a pentagonal-star multiply twinned diamond particle discovered in a natural diamond in 1963 [75]—showed a cyclic 5-fold twin of ~100 μm formed by five octahedral units, accommodating the 7°20′ angular deficit through the polycrystalline material at the core. Subsequent work identified analogous morphologies in synthetic CVD diamonds, including ~5 μm decahedral Wulff polyhedra [77] and nearly ideal ~1 μm decahedra, whose X-ray diffraction patterns confirm symmetric accommodation of the 5-fold 7°20′ mismatch among twin units and boundaries [77]. Additional findings comprise pentagonal-star twins in artificial diamonds [97], ~1 mm star-shaped twins in natural borts from Tortiya [76], and ~600 μm cyclic decahedral needle-shaped crystals in synthetic diamond grits characterized by X-ray, optical, and SEM techniques [93].

More recent hot-filament CVD studies have produced high-quality micro- and nanostructured diamonds—including regular and star-decahedral MTPs—with effective dimensions up to ~4 μm [96], consistent with models that propose a hexacyclo [5.5.1.1^2,6^.1^8,12^.0^3,11^.0^5,9^]pentadecane (–C_15_–) core as an embryonic structural motif. Collectively, these observations show that concave or dimpled vertices are common across size regimes, reflecting relaxation of the intrinsic 5-fold angular misfit through localized defects, stacking-fault lamellae, or minor truncations along {111} or {100} facets.

To understand the unique features of these nanodiamonds, it is important to consider the concepts of crystal twinning and multiple twinning [98,99]. Twinning arises when two or more crystal segments share a subset of lattice points in a symmetrical arrangement. In the case of multiple twinning, several such segments are coherently joined, producing intricate internal architectures that can markedly affect the material’s overall properties. For nanodiamonds, the {111} facets of bulk cubic diamonds (F*d3m* space group) [100] and the basal planes of hexagonal diamonds (P*6_3_*/*mmc* space group) [101] are of particular relevance. The {111} facets of cubic diamonds are energetically favored and frequently participate in the formation of twin boundaries, including multiply twinned particles (MTPs).

In contrast to infinite sp^3^ carbon lattices—cubic or hexagonal—closed-shell multiply twinned nanodiamond clusters have a finite size [102], naturally terminating at the outer surface and inherently precluding the extension of a three-dimensional periodic lattice. Despite this, they preserve characteristic local motifs inherited from the diamond lattice, including tetrahedral sp^3^ coordination and ordered {111} facets, which confer discrete long-range orientational order even in the absence of translational periodicity [102]. Consequently, these entities belong to the class of aperiodic finite solids, where structural order is governed not by infinite lattice vectors but by rotational, inversion, and reflection symmetries centered within the particle. Perhaps the most comprehensive collection of publications on the experimental and theoretical study of the structure and properties of aperiodic multiply twinned diamonds with pentagonal symmetry can be found in the extensive review [102].

In contrast to periodic diamond crystals, multiply twinned nanodiamonds with typical dimensions form through the assembly of several crystallographically coherent tetrahedral or octahedral subdomains joined across twin interfaces, most commonly along {111} planes [103,104]. The resulting closed-shell structures—for example, 5-fold twinned decahedral or 20-fold twinned icosahedral analogs—exhibit central symmetry point groups but inherently frustrate the periodic tiling of space. This geometric frustration produces a non-periodic yet highly ordered internal arrangement, characteristic of 0D nanometer-sized aperiodic solids. Their structural integrity is strengthened by the stability of the {111} facets in sp^3^ carbon [105], and the twin boundaries often incorporate single-layer hexagonal diamond (lonsdaleite-type) interfaces, as experimentally observed in natural, closed-shell twinned diamonds and reproduced in atomistic simulations [106,107,108]. A very typical example is the closed-shell stellar diamond reported in 1932 [78], demonstrating that multiply twinned morphology is not restricted to the nanoscale but reflects a universal geometric constraint of joining tetrahedral diamond subcrystals.

The aperiodic character of such finite particles is further emphasized by the fact that no extension of the interior structure can generate a periodic three-dimensional lattice without disrupting the twin network. Thus, the multiplicity of twinning replaces translational periodicity as the principal organizing rule: the core symmetry is defined exclusively by finite rotational groups (most commonly 2-, 3-, and 5-fold axes depending on twinning geometry), combined with central inversion symmetry for icosahedral particles. As a result, these nanodiamonds behave as genuine 0D centrally symmetric crystalline clusters rather than fragments of a bulk diamond. Similar multiply twinned closed clusters are also known for sp^3^-bonded silicon, germanium [109,110,111], and gold [112,113,114,115], as well as for PtFe_1.2_ nanoparticles [116], extending the concept beyond carbon.

**Figure 5:**
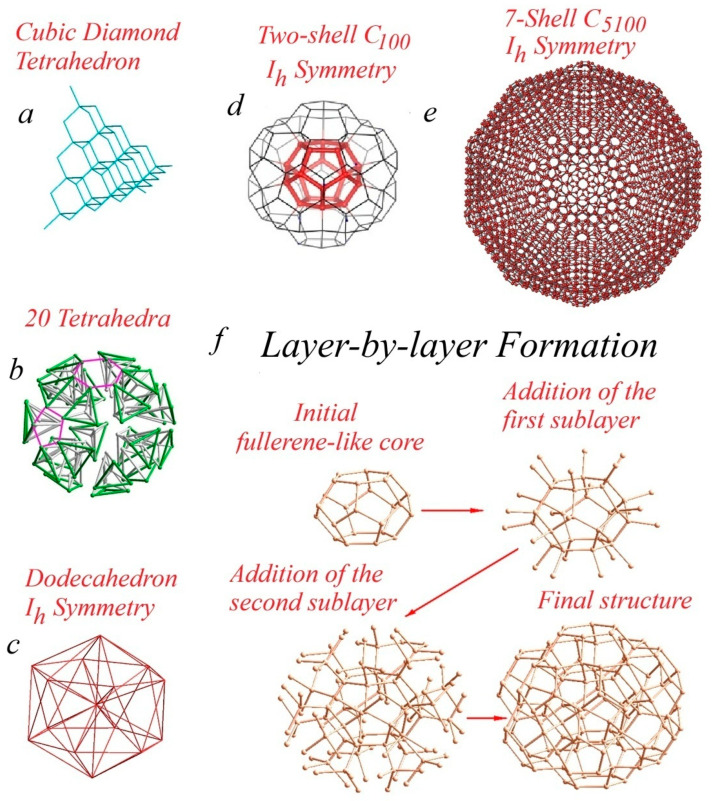
Structure and formation schematics of icosahedral closed-shell multiply twinned nanodiamonds. (**a**) Initial cubic diamond tetrahedron, (**b**) 20 cubic diamond tetrahedra arranged in icosahedron pattern, (**c**) resulting perfect icosahedron of *I_h_* symmetry, (**d**) two-shell C_100_ MTP of *I_h_* symmetry, (**e**) 7-hell C_5100_ MTP of *I_h_* symmetry, and (**f**) layer-by-layer schematic of closed-shell diamond MTP formation.

To understand the structure and properties of icosahedral symmetry MTPs formed through the fusion of twenty tetrahedral subunits (Figure 5a–c) of the cubic diamond lattice, it is useful to examine the symmetry and geometry of finite regular polyhedra—decahedra and icosahedra. The first well-founded proposal regarding the embryonic structure and symmetry of diamond MTPs was put forward by Matsumoto and Matsui [77]. It was suggested that the polycyclic (-C_15_-)_n_ hexacyclo [5.5.1.1^2,6^.1^8,12^.0^3,11^.0^5,9^]pentadecane framework and the fullerene-like dodecahedron C_20_ may serve as structural embryos for closed-shell, Wulff-type decahedral clusters and icosahedral diamond MTPs, respectively.

Theoretical models of icosahedral and decahedral MTPs have been developed based on high-resolution transmission electron microscopy (HRTEM) images [81]. These analyses indicate that the 5-fold structures closely resemble those found in metallic clusters. It is necessary to note that HRTEM images of decahedral particles reveal the presence of heptagonal atomic rings. According to Refs. [77,82,83,86,87,117,118], the formation of diamond MTPs with diverse morphologies is controlled by twinning mechanisms originating from stacking faults during nucleation on {111} surfaces, which are accompanied by reorientation of the crystallographic axes [88].

The foundation of structural analysis lies in the mathematics of three core shapes. The first two are a dual pair, the icosahedron (20 triangles, 12 vertices) and the dodecahedron (12 pentagons, 20 vertices), both exhibiting perfect icosahedral symmetry. The third is the distinct pentagonal decahedron, a structure formed by two pentagonal pyramids joined at their base.

**Figure 6:**
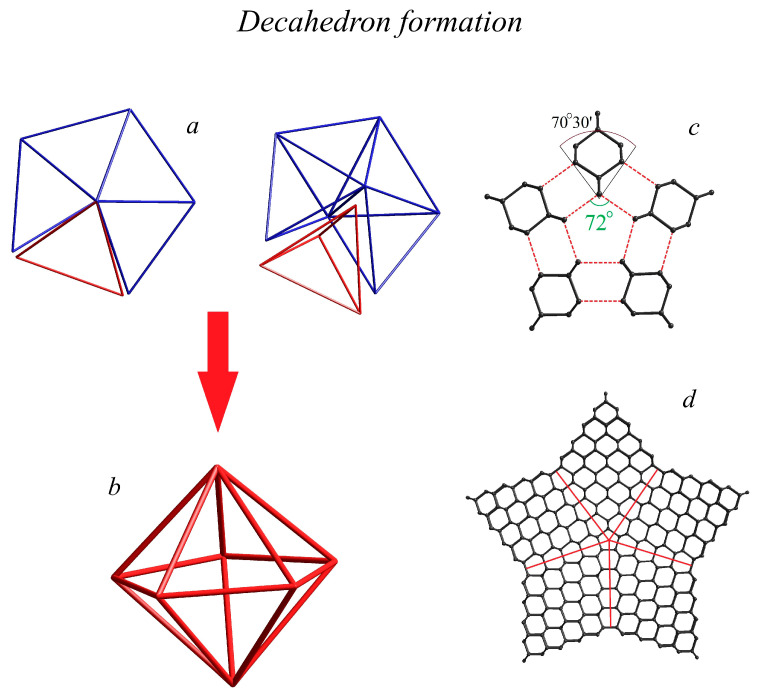
(**a**) Top and perspective views of five tetrahedra assembled into a perfect decahedron (pentagonal bipyramid). The accumulated angular gap of 7°20′, arising from the mismatch between the five dihedral angles of a regular pentagon (72° each) and the five dihedral angles between facets of a regular tetrahedron (70°32′), is clearly visible. (**b**) Ideal perfect decahedron. (**c**) Top view of five C_10_ {110}-oriented cubic diamond fragments (corresponding to the carbon skeletons of adamantane molecules) with the relevant interfacial angles indicated. Fusion of internal five carbon atoms forms a C_5_ base of C_15_ hexacyclo [5.5.1.1^2,6^.1^8,12^.0^3,11^.0^5,9^]pentadecane, which constitutes the core of diamond multiply twinned particles (DMTPs). (**d**) Atomistic model (top view) of a DMTP. The boundaries between the cubic diamond fragments are highlighted by red solid lines. The interfaces between adjacent cubic fragments consist of a single layer of a hexagonal diamond.

A perfect decahedron is formed by the fusion of five slightly distorted, symmetrically equivalent cubic diamond tetrahedra (Figure 6a,b). Since the dihedral angle of 72° between equivalent fragments is 1°28′ larger than the dihedral angle between the facets of the regular tetrahedron (70°32′) fusion of five cubic diamond tetrahedra introduces an accumulated, equally distributed structural stress and lattice distortion of 7°20′ (Figure 6c). Either decahedral or star-shaped diamond decahedral MTPs (DMTP, DMTP/*s*) are formed by the fusion of five diamond tetrahedra/trigonal bipyramids (Figure 6d), respectively, through the {111} facets. For both finite-sized DMTP and DMTP/s atomic lattices, the polycyclic (-C_15_-)_n_ hexacyclo [5.5.1.1^2,6^.1^8,12^.0^3,11^.0^5,9^]pentadecane central core is formed by the fusion of five {110} tetragonal cubic diamond fragments. This forms a pentagonal symmetry central channel with two C_5_ fragments above and below the symmetry plane. The asymptotical expansion of the effective transverse MTP dimension transforms the pentagonal (-C_15_-)_n_ hexacyclo [5.5.1.1^2,6^.1^8,12^.0^3,11^.0^5,9^]pentadecane channel into a 5-fold rotation axis.

Geometrically, a perfect icosahedron is an assembly of twenty tetrahedra (Figure 5), with minor structural distortions caused by the slight mismatch of dihedral angle tetrahedral fragments (70°32′) and the angle of pentagonal rotation (72°). When this principle is applied to diamond synthesis, it leads to icosahedral multiply twinned particles (IMTPs). These IMTPs are created by joining twenty tetrahedral fragments of cubic diamonds on their matching {111} facets, a process that generates internal structural stress and lattice distortion shared uniformly by all tetrahedra. The focal point of this assembly is a central cage of twenty carbon atoms (dodecahedrane, C_20_), which exhibits perfect icosahedral symmetry. The junctions between the fused tetrahedra at this core take the form of a hexagonal diamond. The central C_20_ forms a rather large fullerene-like cavity [119] in the center. Each dodecahedrane pentagonal face conceives a pentagonal channel with central polycyclic (-C_15_-)_n_ poly-hexacyclo [5.5.1.1^2,6^.1^8,12^.0^3,11^.0^5,9^]pentadecane formed by the fusion of five $\langle 110\rangle$-oriented tetragonal neighbors.

The radiating, shell-by-shell packing of spheres in a rigid icosahedron—modeled on the densest “pile-of-cannonballs” configuration (density *ρ*_cp_)—was formalized by Mackay [120]. Owing to the composition of a regular icosahedron from 12 decahedral units, this model also applies to the symmetry and structure of decahedral sphere packings. Maximum density for these decagonal packings is achieved by distorting the tetrahedral units, increasing the length of their 5-fold common edge by a factor of 3sinπ5=1.018 [121], resulting in a density of 0.991 *ρ*_cp_.

Three tetrahedral edges are stretched by a factor of 85+5=1.051 [122], with the solid angle at the center of the icosahedron, φIc=4π20sr=0.628sr, visibly larger than the solid angle at a regular tetrahedron vertex, φTv=arccos13−π=4π22.8sr=0.551sr. The accumulated solid-angle stretching is $\left(0.628sr-0.551sr\right)\times 20=1.54sr$, with modest 0.077*sr* stretching per each tetrahedron unit. For icosahedral packing, the icosahedron density can be expressed in terms of *ρ*_cp_, namely, ρI=5ρcp25+5=0.929ρcp.

To avoid strain, each tetrahedral fragment should follow rhombohedral symmetry with the angle $\alpha =arccos\left(\frac{1}{\sqrt{5}}\right)=63{}^{\circ}26^{\prime }=1.1072rad.$ This angle corresponds to the angle between adjacent 5-fold axes of an icosahedron equivalent to ca=343+5=2.267. For perfect central cubic packing, one can write the following: α=60°=π3sr and ca=6=2.45. This restriction causes visible mechanical stress to be symmetrically distributed among the entire IMTP.

In 1993, Zeger and Kaxirai first proposed structures consisting of sp^3^-hybridized carbon atoms, featuring a 3-fold-coordinated surface and a 4-fold-coordinated bulk [89,123]. Independently, Zhao et al. extended this concept to silicon, proposing analogous icosahedral clusters in 2004 [124]. The underlying structural model for both systems posits a cubic diamond lattice architecture. This framework is formed by the fusion of twenty identical tetrahedral units along their equivalent $\langle 111\rangle$ planes, creating twin interfaces of hexagonal diamonds between the cubic fragments.

The central, highly symmetrical core of an IMTP consists of 20 carbon atoms derived from the vertices of its 20 tetrahedral building blocks. As growth proceeds outward, covalently bonded $\langle 111\rangle$ diamond planes form a series of concentric shells. The topology of these shells is equivalent to that of icosahedral fullerenes with (n, 0) chirality, generating a sequence of potential cages like C_20_, C_80_, C_180_, C_320_, C_500_, C_720_, C_980_, C_1280_, C_1620_, C_2000_, and larger members or analogous silicon-based structures [125,126,127,128,129].

At the core of the lattice resides a regular, achiral (1,0) C_20_ dodecahedrane with perfect icosahedral symmetry. Its 20 4-fold-coordinated carbon atoms form 12 symmetric pentagons via sp^3^ bonds with internal angles of 108°. This angle is close to the ideal tetrahedral bond angle (109.471°), but the slight mismatch of 1.471° can generate cumulative structural stress as the lattice extends through the addition of concentric carbon shells.

Figure 5d presents the simplest closed-shell icosahedral nanodiamond cluster, comprising two concentric shells: a central C_20_ dodecahedron and an outer C_80_ shell. Each carbon atom (red) in the central C_20_ core forms four sp^3^ bonds: three bonds participate in the three fused pentagons that constitute the dodecahedron, while the fourth bond connects covalently to the surrounding C_80_ shell via twenty linkage sites.

The C_80_ shell itself has a dual structure: an inner subshell of 20 atoms (yellow) bonded directly to the core and an outer subshell of 60 surface atoms (black). Each atom in this outer C_60_ subshell has three inward-directed sp^3^ bonds and one outward-facing dangling bond.

The intermediate C_20_ (yellow) atoms each form three sp^3^ bonds to the outer C_60_ subshell. The dangling bonds on the surface can be passivated, for instance, by hydrogen, yielding a hydrogenated C_100_H_60_ cluster of icosahedral symmetry. The sixty surface atoms are arranged into twelve pentagonal vertices. Expanding this lattice by adding further concentric closed shells leads to the development of characteristic external $\langle 111\rangle$ cubic diamond facets.

For aperiodic icosahedral nanodiamonds, crystal momentum *k* is not a good quantum number because of the absence of translational long-range order. Despite this, time-reversal symmetry remains well defined and enforces Kramers degeneracy, ensuring that each electronic state is at least 2-fold degenerated in the absence of magnetic order [130,131,132]. Importantly, the centrosymmetric icosahedral point group *I_h_* possesses a global inversion center. Time-reversal symmetry relates states according to E(k,↑)=E(−k,↓), while space-inversion symmetry leaves the spin unchanged and imposes E(k,s)=E(−k,s). When both symmetries are present, these relations combine to yield the fundamental condition E(k,↑)=E(k,↓), guaranteeing exact spin degeneracy even in the presence of appreciable spin–orbit coupling [133,134]. Consequently, perfect multiply twinned icosahedral nanodiamonds exhibit symmetry-protected electronic degeneracy, characteristic of a confined, zero-dimensional, centrosymmetric aperiodic crystalline solid rather than that of conventional non-centrosymmetric nanostructures.

The electronic properties of perfect icosahedral nanodiamonds fundamentally contrast with those of conventional three-dimensional quasicrystals. Although quasicrystals may exhibit global icosahedral rotational symmetry, they generally lack exact inversion symmetry at the level of the full atomic structure, owing to their aperiodic long-range order and the presence of phason degrees of freedom [135]. As a result, inversion cannot be treated as a strict symmetry operation acting on electronic states, and spin degeneracy is protected solely by time-reversal symmetry. In such systems, spin–orbit coupling combined with local inversion asymmetry can lift the spin degeneracy away from special symmetry points, giving rise to Rashba- or Dresselhaus-like splittings even in the absence of magnetism [136,137]. Consequently, although both perfect icosahedral nanodiamonds and three-dimensional quasicrystals are non-translationally invariant solids, their electronic structures differ qualitatively: the former can retain exact Kramers-paired, spin-degenerate spectra due to the coexistence of time-reversal and inversion symmetries in the *I_h_* point group, whereas the latter generally cannot rely on inversion symmetry and therefore exhibit more complex, symmetry-reduced electronic degeneracy patterns.

It is important to emphasize that this conclusion does not extend to the electronic structure and spin states of decahedral multiply twinned nanodiamonds. Perfect decahedra belong to the *D*_5*h*_ point group, which lacks an inversion center. As a consequence, although time-reversal symmetry is preserved in the absence of magnetic fields, the absence of spatial inversion removes the protection of spin degeneracy that characterizes centrosymmetric systems. In contrast to icosahedral multiply twinned particles—where the coexistence of time-reversal symmetry and effective inversion symmetry enforces Kramers-degenerate electronic states—the electronic levels in decahedral twins may undergo spin splitting driven by spin–orbit coupling in a non-centrosymmetric crystal field. The presence of pentagonal symmetry combined with hexagonal twin boundaries can therefore enable spin polarization, Rashba-like spin textures, and potentially unconventional magnetic responses, even in nominally sp^3^-bonded carbon systems. These features make decahedral nanodiamonds fundamentally distinct from their icosahedral counterparts, with respect to spin physics, and highlight the critical role of point-group symmetry in determining the spin structure of multiply twinned nanoparticles.

Boron-based icosahedral quasicrystals provide an instructive example for highlighting the structural distinctions between icosahedral multiply twinned diamond nanoparticles and conventional three-dimensional covalent quasicrystals. A variety of nonmetallic boron quasicrystals exhibiting icosahedral symmetry or decagonal pentagonal bipyramidal motifs have been experimentally identified. These structures are built from B_12_ or larger B_66_ (B_12_(B_12_)_12_) dodecahedral clusters with intrinsic icosahedral symmetry and may be stabilized with the incorporation of dopant ions such as O or Na (see, for example, Refs. [138,139]). The corresponding parent crystalline phase, boron suboxide, adopts a rhombohedral lattice that closely approaches the geometric conditions required for the formation of icosahedrally multiply twinned particles. The small B_12_ pentagonal dodecahedral clusters, possessing icosahedral symmetry, fulfill the Mackay packing criterion [140], thereby enabling the emergence of decahedral pentagonal bipyramids with ten triangular facets, as well as dodecahedral, icosahedral, and rhombic triacontahedral morphologies with overall icosahedral symmetry [141,142,143,144,145].

Boron suboxide B_6_O [138] is a member of the rhombohedral family of boron-rich compounds [146,147]. It was synthesized within a soft, water-soluble boron–oxide matrix, yielding orange-red grains approximately 30 μm in size that exhibit an almost ideal icosahedral morphology. Selected-area electron diffraction patterns of the B_6_O icosahedral particles [65] clearly reveal 5-fold symmetry. In the corresponding X-ray diffraction data, intense reflections associated with the internal pentagonal motif are observed at 1/0.436 nm^−1^ (1/d(100)_r_) and 1/0.37 nm^−1^ (1/d(110)_r_).

Powder X-ray diffraction data for icosahedral boron α-suboxide particles [138] exhibit characteristic rhombohedral diffraction patterns [138,139,140,141,142,143,144,145,146,147,148,149,150] consistent with the presence of nearly perfect B_6_O icosahedra. Scanning electron microscopy reveals well-defined re-entrant angles at the particle vertices, a hallmark of twinned morphologies. The multiply twinned nature of the α-rhombohedral B_6_O icosahedral particles is further unambiguously confirmed by transmission electron microscopy, which shows twin interfaces characterized by an interplanar spacing of 4.4 Å.

The atomic framework of β-rhombohedral boron suboxide is built from nanoscale B_12_ pentagonal dodecahedral clusters possessing icosahedral symmetry, which assemble into macroscopic B_6_O quasicrystals exhibiting perfect icosahedral symmetry but lacking translational periodicity. In the β-B_6_O allotrope [151,152], the lattice is composed of B_12_ (B_12_)_12_ super-icosahedra, consisting of a central B_12_ icosahedron surrounded by twelve B_12_ structural units. Adjacent icosahedra are interconnected through oxygen ions.

In contrast to intermetallic compounds and β-rhombohedral boron suboxide, which display characteristic quasicrystalline diffraction patterns with pentagonal symmetry, multiply twinned boron α-suboxide particles as well as icosahedral and dodecahedral nano- and meso-diamonds constitute fundamentally different classes of non-translationally invariant solids, distinguished by point-reflection symmetry. The presence of potential barriers between either five (in dodecahedral particles) or twenty (in icosahedral particles) cubic subunits, arising from hexagonal twin boundaries, is expected to give rise to strongly correlated electronic states and to induce effective, mimicked quantum confinement in both nano- and meso-diamonds.

## 4. Conclusions

Low-dimensional aperiodic crystalline solids arise from multiple, fundamentally distinct mechanisms that go beyond the classical quasicrystal paradigm. While three-dimensional quasicrystals owe their aperiodicity to higher-dimensional crystallographic projections, reduced-dimensional systems exhibit additional sources of periodicity breakdown rooted in thermodynamic instability, topological constraints, and finite-size effects, in particular, caused by the elimination of some force constants. Multiply twinned nanoparticles represent a unique class of zero-dimensional aperiodic solids in which geometric frustration and internal stress preclude translational order despite local crystalline bonding. The symmetry of these finite clusters plays a decisive role in their electronic structure: icosahedral MTPs preserve spin degeneracy through the combined action of time-reversal symmetry and high orientational symmetry, whereas decahedral MTPs lack inversion symmetry and may exhibit spin splitting and spin polarization. These findings highlight the intimate connection between dimensionality, symmetry, and electronic degrees of freedom in aperiodic solids and underscore the importance of finite-size and symmetry effects in nanoscale materials.

Although aperiodicity is fundamentally a crystallographic and physical property rather than a design objective *per se*, it can nonetheless be regarded as a structural principle that enables functionalities not accessible in periodic systems. In low-dimensional systems, aperiodic order has been exploited to engineer electronic and vibrational spectra, most notably through the appearance of critical states and spectral gaps that are robust against local perturbations. One-dimensional and two-dimensional aperiodic lattices, such as quasiperiodic and defect-engineered tilings, could been employed to achieve wave localization, broadband phononic and photonic filtering, and tunable transport properties, finding applications in photonic quasicrystals, metamaterials, and surface-supported nanostructures. In these systems, aperiodicity allows for the decoupling of symmetry from translational periodicity, enabling control over degeneracies and mode distributions beyond the constraints of conventional Bloch theory.

## Data Availability

No new data were created or analyzed in this study. Data sharing is not applicable to this article.

## References

[1] Levine D., Steinhardt P.J. (1984). Quasicrystals: A new class of ordered structures. Phys. Rev. Lett..

[2] Levine D., Steinhardt P.J. (1986). Quasicrystals. I. Definition and structure. Phys. Rev. B.

[3] Elser V., Henley C.L. (1985). Crystal and quasicrystal structures in Al–Mn–Si alloys. Phys. Rev. Lett..

[4] Noya E.G., Wong C.K., Llombart P., Doye J.P.K. (2021). How to design an icosahedral quasicrystal through directional bonding. Nature.

[5] Penrose R. (1974). The role of aesthetics in pure and applied mathematical research. Bull. Inst. Math. Appl..

[6] Penrose R. (1979). Pentaplexity: A class of nonperiodic tilings of the plane. Math. Intell..

[7] Dubois J.-M. (2012). Properties and applications of quasicrystals and complex metallic alloys. Chem. Soc. Rev..

[8] Scherrer W. (1946). Die Einlagerung eines regulären Vielecks in ein Gitter. Elem. Der Math..

[9] Bamberg J., Cairns G., Kilminster D. (2003). The crystallographic restriction, permutations, and Goldbach’s conjecture. Am. Math. Mon..

[10] Bončina T., Zupanič F. (2025). Metallography of quasicrystals in Al-alloys. Materials.

[11] Steurer W. (1994). The structure of quasicrystals. Mater. Sci. Forum..

[12] Louzguine-Luzgin D.V., Inoue A. (2008). Formation and properties of quasicrystals. Annu. Rev. Mater. Res..

[13] Landau L.D., Lifshitz E.M. (2005). Course of Theoretical Physics, Vol. 5: Statistical Physics, Part 1.

[14] Mermin N.D. (1968). Crystalline order in two dimensions. Phys. Rev..

[15] Nelson D.R., Piran T., Weinberg S. (2004). Statistical Mechanics of Membranes and Surfaces.

[16] Nelson D.R., Peliti L. (1987). Fluctuations in membranes with crystalline and hexatic order. J. Physique.

[17] Le Doussal P., Radzihovsky L. (1992). Self-consistent theory of polymerized membranes. Phys. Rev. Lett..

[18] Mermin N.D., Wagner H. (1966). Absence of ferromagnetism or antiferromagnetism in one- or two-dimensional isotropic Heisenberg models. Phys. Rev. Lett..

[19] Hohenberg P.C. (1967). Existence of long-range order in one and two dimensions. Phys. Rev..

[20] Zakharchenko K.V., Katsnelson M.I., Fasolino A. (2009). Atomistic simulations of structural and thermodynamic properties of bilayer graphene. Phys. Rev. Lett..

[21] Los J.H., Zakharchenko K.V., Katsnelson M.I. (2009). Scaling properties of flexible membranes from atomistic simulations: Application to graphene. Phys. Rev. B.

[22] Fasolino A., Los J.H., Katsnelson M.I. (2007). Intrinsic ripples in graphene. Nat. Mater..

[23] Meyer J.C., Geim A.K., Katsnelson M.I., Novoselov K.S., Booth T.J., Roth S. (2007). The structure of suspended graphene sheets. Nature.

[24] Girit C., Meyer J.C., Erni R., Roseli M.D., Kisielowski C., Yang L., Park C.H., Crommie M.F., Cohen M.L., Loie S.G. (2009). Graphene at the Edge: Stability and Dynamics. Science.

[25] Bao W., Miao F., Chen Z., Zhang H., Jang W., Dames C., Lau C.N. (2009). Controlled ripple texturing of suspended graphene and ultrathin graphite membranes. Nat. Nanotechnol..

[26] Tapasztó L., Dumitrică T., Kim S.J., Nemes-Incze P., Hwang C., Biró L.P. (2012). Breakdown of continuum mechanics for nanometre-wavelength rippling of graphene. Nat. Phys..

[27] Larach S., Shrader R.E. (1956). Electroluminescence from boron nitride. Phys. Rev..

[28] Elias D.C., Nair R.R., Mohiuddin T.M.G., Morozov S.V., Blake P., Halsall M.P., Ferrari A.C., Boukhalv D.W., Katsnelson M.I., Geim A.K. (2009). Novoselov, Control of graphene’s properties by reversible hydrogenation: Evidence for graphane. Science.

[29] Ketabi N., Tolhurst T.M., Leedahl B., Liu H., Li Y., Moewes A. (2017). How functional groups change the electronic structure of graphdiyne: Theory and experiment. Carbon.

[30] Jia Z., Li Y., Zuo Z., Liu H., Huang C., Li Y. (2017). Synthesis and properties of 2D carbon—Graphdiyne. Acc. Chem. Res..

[31] Desyatkin V.G., Martin W.B., Aliev A.E., Chapman N.E., Fonseca A.F., Galvão D.S., Miller E.R., Stone K.H., Wang Z., Zakhidov D. (2022). Scalable synthesis and characterization of multilayer γ-graphyne, new carbon crystals with a small direct band gap. J. Am. Chem. Soc..

[32] Liu X., Cho S.M., Lin S., Chen Z., Choi W., Kim Y.-M., Yun E., Baek E.H., Ryu D.H., Lee H. (2022). Constructing two-dimensional holey graphyne with unusual annulative π-extension. Matter.

[33] Li Y., Zhang J., Wang Q., Jin Y., Huang D., Cui Q., Zou G. (2010). Nitrogen-rich carbon nitride hollow vessels: Synthesis, characterization, and their properties. J. Phys. Chem. B.

[34] Lee J.S., Wang X.Q., Luo H.M., Dai S. (2010). Fluidic Carbon Precursors for Formation of Functional Carbon under Ambient Pressure Based on Ionic Liquids. Adv. Mater..

[35] Chernozatonskii L.A., Sorokin P.B., Kvashnin A.G., Kvashnin D.G. (2009). Diamond-like C2H nanolayer, diamane: Simulation of the structure and properties. JETP Lett..

[36] Bakharev P.V., Huang M., Saxena M., Lee S.W., Joo S.H., Park S.O., Dong J., Camacho-Mojica D.C., Jin S., Kwon Y. (2020). Chemically induced transformation of CVD-grown bilayer graphene into fluorinated single-layer diamond. Nat. Nanotechnol..

[37] O’Hare A., Kusmartsev F.V., Kugel K.I., Stable A. (2012). “Flat” Form of Two-Dimensional Crystals: Could Graphene, Silicene, Germanene Be Minigap Semiconductors?. Nano Lett..

[38] Avramov P. (2023). Topology conservation theorem, quantum instability and violation of subperiodic symmetry of complex low-dimensional lattices. Acta Crystallogr..

[39] Avramov P.V., Kuklin A.V. (2022). Topological and Quantum Stability of Low-Dimensional Crystalline Lattices with Multiple Nonequivalent Sublattices. New J. Phys..

[40] Taskaev S., Skokov K., Khovaylo V., Donner W., Faske T., Dudorov A., Gorkavyi N., Muratov D.S., Savosteenko G., Dyakonov A. (2022). Exotic carbon microcrystals in meteoritic dust of the Chelyabinsk superbolide: Experimental investigations and theoretical scenarios of their formation. Eur. Phys. J. Plus.

[41] Kuklin A., Ågren H., Avramov P.V. (2020). Structural stability of single-layer PdSe_2_ with pentagonal puckered morphology and its nanotubes. Phys. Chem. Chem. Phys..

[42] Avramov P., Demin V., Luo M., Choi C.H., Sorokin P.B., Yakobson B., Chernozatonskii L. (2015). Translation Symmetry Breakdown in Low-Dimensional Lattices of Pentagonal Rings. J. Phys. Chem. Lett..

[43] Avramov P.V., Fedorov D.G., Sorokin P.B., Sakai S., Entani S., Ohtomo M., Matsumoto Y., Naramoto H. (2012). Intrinsic Edge Asymmetry in Narrow Zigzag Hexagonal Heteroatomic Nanoribbons Causes their Subtle Uniform Curvature. J. Phys. Chem. Lett..

[44] Gauss G.F. (1827). Disquisitiones Generales Circa Superficies Curvas.

[45] do Carmo M.P. (1976). Differential Geometry of Curves and Surfaces.

[46] Weisstein E.W. Gauss’s Theorema Egregium, MathWorld—A Wolfram Web Resource. https://mathworld.wolfram.com/TheoremaEgregium.html.

[47] Neto A.H.C., Guinea F., Peres N.M.R., Novoselov K.S., Geim A.K. (2009). The electronic properties of graphene. Rev. Mod. Phys..

[48] Lee J.M. (2018). Introduction to Riemannian Manifolds, Graduate Texts in Mathematics.

[49] Cheeger J., Müller W., Schrader R. (1984). On the curvature of piecewise flat spaces. Commun. Math. Phys..

[50] Kroto H., Heath J.R., O’Brien S.C., Curl R.F. (1985). Smalley C60: Buckminsterfullerene. Nature.

[51] Prinz V.Y., Seleznev V.A., Gutakovsky A.K., Chehovskiy A.V., Preobrazhenskii V.V., Putyato M.A., Gavrilova T.A. (2000). Free-standing and overgrown InGaAs=GaAs nanotubes, nanohelices and their arrays. Physica E.

[52] Kotakoski J., Krasheninnikov A.V., Kaiser U., Meyer J.C. (2011). From Point Defects in Graphene to Two-Dimensional Amorphous Carbon. Phys. Rev. Lett..

[53] Kotakoski J., Meyer J.C., Kurasch S., Santos-Cottin D., Kaiser U., Krasheninnikov A.V. (2011). Stone-Wales-type transformations in carbon nanostructures driven by electron irradiation. Phys. Rev. B.

[54] Kuzubov A.A., Avramov P.V., Tomilin F.N., Ovchinnikov S.G. (2001). Theoretical investigation of toroidal forms of carbon and their endohedral derivatives with Li ions. Phys. Solid State.

[55] Grünbaum B., Shephard G.C. (1987). Tilings and Patterns.

[56] Yadav T.P., Woellner C.F., Sinha S.K., Sharifi T., Apte A., Mukhopadhyay N.K., Srivastava O.N., Vajtai R., Galvao D.S., Tiwary C.S. (2018). Liquid exfoliation of icosahedral quasicrystals. Adv. Funct. Mater..

[57] Yadav T.P., Woellner C.F., Sharifi T., Sinha S.K., Qu L.-L., Apte A., Mukhopadhyay N.K., Srivastava O.N., Vajtai R., Galvão D.S. (2020). Extraction of Two-Dimensional Aluminum Alloys from Decagonal Quasicrystals. ACS Nano.

[58] Ngoc B.P., Geantet C., Aouine M., Bergeret G., Marlin S.R.S. (2008). Quasicrystal derived catalyst for steam reforming of methanol. Int. J. Hydrogen Energy.

[59] Kameoka S., Tanabe T., Tsai A.P. (2004). Al-Cu-Fe quasicrystals for steam reforming of methanol: A new form of copper catalysts. Catal. Today.

[60] Tanabe T., Kameoka S., Tsai A.P. (2010). Microstructure of leached Al-Cu-Fe quasicrystal with high catalytic performance for steam reforming of methanol. Appl. Catal. A Gen..

[61] Tanabe T., Kameoka S., Tsai A.P. (2011). Evolution of microstructure induced by calcination in leached Al-Cu-Fe quasicrystal and its effects on catalytic activity. J. Mater. Sci..

[62] Pandey S.K., Bhatnagar A., Mishra S.S., Yadav T.P., Shaz M.A., Srivastava O.N. (2017). Curious Catalytic Characteristics of Al–Cu– Fe Quasicrystal for De/Rehydrogenation of MgH_2_. J. Phys. Chem. C.

[63] Yamamoto A. (2008). Software package for structure analysis of quasicrystals. Sci. Technol. Adv. Mater..

[64] Kittel C. (2005). Introduction to Solid State Physics.

[65] Zallen R. (2004). The Physics of Amorphous Solids.

[66] van Smaalen S. (2007). Incommensurate Crystallography, IUCr Monographs.

[67] Grimm U., Scheffer M. (2001). Incommensurate Crystals and Quasicrystals. Encyclopedia of Physical Science and Technology.

[68] Suck J.-B. (2005). Lattice Dynamics: Aperiodic Crystals. Encyclopedia of Condensed Matter Physics.

[69] Stampfli P. (1986). A dodecagonal quasiperiodic lattice in two dimensions. Helv. Phys. Acta.

[70] Koren E., Duerig U. (2016). Superlubricity in quasicrystalline twisted bilayer graphene. Phys. Rev. B.

[71] Ahn S.J., Moon P., Kim T.-H., Kim H.-W., Shin H.-C., Kim E.H., Cha H.W., Kahng S.-J., Kim P., Koshino M. (2018). Dirac electrons in a dodecagonal graphene quasicrystal. Science.

[72] Santos J.M.B.L.D., Peres N.M.R., Neto A.H.C. (2007). Graphene bilayer with a twist: Electronic structure. Phys. Rev. Lett..

[73] Kraus Y.E., Ringel Z., Zilberberg O. (2013). Four-dimensional quantum Hall effect in a two-dimensional quasicrystal. Phys. Rev. Lett..

[74] Hu S., Sun J., Du X., Tian F., Jiang L. (2008). The Formation of Multiply Twinning Structure and Photoluminescence of Well-Dispersed Nanodiamonds Produced by Pulsed-Laser Irradiation. Diam. Relat. Mater..

[75] Wentorf R.H., Gilman J. (1963). The Art and Science of Growing Crystals.

[76] Casanova R., Simon B., Turco G. (1972). A Repeated Twin in Natural Diamond From Tortiya, Ivory Coast. Am. Mineral..

[77] Matsumoto S., Matsui Y. (1983). Electron Microscopic Observation of Diamond Particles Grown from the Vapor Phase. J. Mater. Sci..

[78] Palache C. (1932). Multiple Twins of Diamond and Sphalerite. Am. Mineral..

[79] Carrington W.A., Hanssen L.M., Snail K.A., Oakes D.B., Butler J.E. (1989). Diamond growth in O_2_ + C_2_H_4_ and O_2_ + C_2_H_2_ flames. Metall. Trans. A.

[80] Ohsumi K., Takase T., Hagiya K., Shimizugawa Y., Miyamoto M., Mitsuda Y., Ohmasa M. (1992). Characterization of 5-μm-sized icosahedral chemical vapor deposited diamond by synchrotron x-ray diffraction with the Laue method. Rev. Sci. Instrum..

[81] Miki-Yoshida M., Rendón L., Tehuacanero S., José-Yacamán M. (1993). Icosahedral, decahedral and single faulted particles obtained from carbon soot. Surf. Sci..

[82] Sunkara M.K., Angus J.C., Hayman C.C., Buck F.A. (1990). Nucleation of diamond crystals. Carbon.

[83] Mani R.C., Sunkara M.K. (2003). Kinetic faceting of multiply twinned diamond crystals during vapor phase synthesis. Diam. Relat. Mater..

[84] Iakoubovskii K., Adriaenssens G.J. (1999). Photoluminescence in CVD Diamond Films. Phys. Status Solidi (a).

[85] Butler J.E., Windischmann H. (1998). Developments in CVD-Diamond Synthesis During the Past Decade. MRS Bull..

[86] Narayan J., Srivatsa A.R., Peters M., Yokota S., Ravi K.V. (1988). On epitaxial growth of diamond films on (100) silicon substrates. Appl. Phys. Lett..

[87] Narayan J., Srivatsa A.R., Ravi K.V. (1989). Mechanism of formation of <110> oriented fivefold microcrystallites in diamond films. Appl. Phys. Lett..

[88] Angus J.C., Buck F.A., Sunkara M.K., Groth T.F., Hayman C.C., Gat R. (1989). Diamond growth at low pressures. MRS Bull..

[89] Zeger L., Kaxiras E. (1993). New Model for Icosahedral Carbon Clusters and the Structure of Collapsed Fullerite. Phys. Rev. Lett..

[90] Breza J.J., Kadlečíková M., Vojs M., Michalka M., Veselý M., Daniš T. (2004). Diamond icosahedron on a TiN-coated steel substrate. Microelectron. J..

[91] Lee S., Glotzer S.C. (2022). Entropically engineered formation of fivefold and icosahedral twinned clusters of colloidal shapes. Nat. Commun..

[92] Song C.W., Lee Y.H., Choi S., Hwang N.-M., Kim K.H. (2019). Formation of pentagonal dimples in icosahedral diamond crystals grown by hot filament chemical vapor deposition: Approach by non-classical crystallization. Coatings.

[93] Pipkin N.J., Davies D.J. (1979). The crystal morphology of cyclic twins in synthetic diamond grits. Philos. Mag. A.

[94] Sunkara M.K. (1993). Monte Carlo Simulation of Diamond Nucleation and Growth. Ph.D. Thesis.

[95] Angus J.C., Argoitia A., Gat R., Li Z., Sunkara M., Wang L., Wang Y. (1993). Chemical vapour deposition of diamond. Philos. Trans. R. Soc. Lond. A.

[96] Wei Q.-P., Ma L., Ye J., Yu Z.-M. (2015). Growth mechanism of icosahedral and other five-fold symmetric diamond crystals. Trans. Nonferrous Met. Soc. China.

[97] Lemmlein G.G., Kliya M.O., Chernov A.A. (1964). Morphological Investigation of Artificial Diamond Crystals. Sov. Phys. Crystallogr..

[98] Nesse W.D. (2000). Introduction to Mineralogy.

[99] Klein C., Hurlbut C.S. (1993). Manual of Mineralogy.

[100] Kobashi K. (2005). Diamond Films: Chemical Vapor Deposition for Oriented and Heteroepitaxial Growth.

[101] Frondel C., Marvin U.B. (1967). Lonsdaleite, a Hexagonal Polymorph of Diamond. Nature.

[102] Melchakova I.A., Oyeniyi G.T., Engelgardt D.R., Polyutov S.P., Avramov P.V. (2024). Pentagonal Symmetry in Aperiodic 0D Multiply-Twinned Nano- and Mezodiamonds. J. Phys. Chem. A.

[103] Danilenko V.V. (2004). On the History of the Discovery of Nanodiamond Synthesis. Phys. Solid State.

[104] Chernozatonskii L.A., Sorokin P.B., Kuzubov A.A., Sorokin B.P., Kvashnin A.G., Kvashnin D.G., Avramov P.V., Yakobson B.I. (2011). Influence of Size Effect on the Electronic and Elastic Properties of Diamond Films with Nanometer Thickness. J. Phys. Chem. C.

[105] Kvashnin A.G., Avramov P.V., Kvashnin D.G., Chernozatonskii L.A., Sorokin P.B. (2017). The Features of Electronic, Mechanical and Electromechanical Properties of Fluorinated Diamond Films of Nanometer Thickness. J. Phys. Chem. C.

[106] Dorignac D., Delclos S., Phillipp F. (2001). Atomic structure of a complex defect configuration in synthetic diamond: A fivefold twin center connected to two high-order grain boundaries. Philos. Mag. B.

[107] Vaughan D.J., Pattrick R.A.D. (1995). Mineralogy: An Introduction to the Study of Minerals and Crystals.

[108] Cullity B.D., Stock S.R. (2001). Elements of X-Ray Diffraction.

[109] Iijima S. (1987). Fine Particles of Silicon. I. Crystal Growth of Spherical Particles of Si. Jpn. J. Appl. Phys..

[110] Iijima S. (1987). Fine Particles of Silicon. II. Decahedral Multiply-Twinned Particles. Jpn. J. Appl. Phys..

[111] Saito Y. (1979). Crystal structure and habit of silicon and germanium particles grown in argon gas. J. Cryst. Growth.

[112] Yamazoe S., Takano S., Kurashige W., Yokoyama T., Nitta K., Negishi Y., Tsukuda T. (2016). Hierarchy of bond stiffnesses within icosahedral-based gold clusters protected by thiolates. Nat. Commun..

[113] Ino S. (1966). Epitaxial Growth of Metals on Rocksalt Faces Cleaved in Vacuum. II. Orientation and Structure of Gold Particles Formed in Ultrahigh Vacuum. J. Phys. Soc. Jpn..

[114] Ogawa S., Ino S. (1969). Formation of Multiply-Twinned Particles in the Nucleation Stage of Film Growth. J. Vac. Sci. Technol..

[115] Komoda T. (1968). Study on the Structure of Evaporated Gold Particles by Means of a High Resolution Electron Microscope. Jpn. J. Appl. Phys..

[116] Jang F.J.-H., Lee E., Park J., Kim G., Hong S., Kwon Y.-U. (2013). Rational syntheses of core-shell Fex@Pt nanoparticles for the study of electrocatalytic oxygen reduction reaction. Sci. Rep..

[117] Bühler J., Prior Y. (2000). Study of morphological behavior of single diamond crystals. J. Cryst. Growth.

[118] Williams B.E., Kong H.S., Glass J.T. (1990). Electron microscopy of vapor phase deposited diamond. J. Mater. Res..

[119] Avramov P.V., Fedorov D.G., Sorokin P.B., Chernozatonskii L.A. (2007). Gordon, Atomic and Electronic Structure of New Hollow-Based Symmetric Families of Silicon Nanoclusters. J. Phys. Chem. C.

[120] Mackay A.L. (1962). A dense non-crystallographic packing of equal spheres. Acta Crystallogr..

[121] Bagley B.G. (1970). Five-Fold Pseudosymmetry. Nature.

[122] O’Keeffe M., Hyde B.G. (1996). Crystal Structures I: Patterns and Symmetry.

[123] Zeger L., Kaxiras E. (1993). Compact Carbon Clusters with Tetrahedral Bonding and Icosahedral Symmetry. Comp. Mater. Sci..

[124] Zhao Y., Kim Y.-H., Du M.-H., Zhang S.B. (2004). First-Principles Prediction of Icosahedral Quantum Dots for Tetravalent Semiconductors. Phys. Rev. Lett..

[125] Savosteenko G., Taskaev S., Avramov P. (2023). Structure and Raman spectra of exotic carbon microcrystals from meteoritic dust of Chelyabinsk superbolide. Nanomaterials.

[126] Goldberg M. (1935). The Isoperimetric Problem for Polyhedra. Tohoku Math. J..

[127] Goldberg M. (1937). A Class of Multi-Symmetric Polyhedra. Tohoku Math. J..

[128] Fowler P.W., Rogers K.M. (2001). Spiral codes and Goldberg representations of icosahedral fullerenes and octahedral analogues. J. Chem. Inf. Comput. Sci..

[129] King R.B., Diudea M.V. (2006). The Chirality of Icosahedral Fullerenes: A Comparison of the Tripling (leapfrog), Quadrupling (chamfering), and Septupling (capra) Transformations. J. Math. Chem..

[130] Kramers H.A. (1930). Théorie générale de la rotation paramagnétique dans les cristaux. Proc. Acad. Sci. Amst..

[131] Wigner E.P. (1959). Group Theory and Its Application to the Quantum Mechanics of Atomic Spectra.

[132] Hasan M.Z., Kane C.L. (2010). Colloquium: Topological insulators. Rev. Mod. Phys..

[133] Dresselhaus M.S., Dresselhaus G., Jorio A. (2008). Group Theory: Application to the Physics of Condensed Matter.

[134] Bradley C.J., Cracknell A.P. (2009). The Mathematical Theory of Symmetry in Solids: Representation Theory for Point Groups and Space Groups.

[135] Steinhardt P.J., Ostlund S. (1987). The Physics of Quasicrystals.

[136] Fu L., Kane C.K. (2007). Topological insulators with inversion symmetry. Phys. Rev. B.

[137] Ishii F., Mizuta Y., Sawahata H., Yamaguchi N. (2019). First-principles study of oxide skyrmion crystal Chern insulator. APS March Meet. Abstr..

[138] Hubert H., Devouard B., Garvie L.A.J., O’Keeffe M., Buseck P.R., Petuskey W.T., McMillan P.F. (1998). Icosahedral packing of B_12_ icosahedra in boron suboxide (B_6_O). Nature.

[139] Naslain R., Matkovitch V.I. (1977). Crystal Chemistry of Boron and of Some Boron-Rich Phases; Preparation of Boron Modifications. Boron and Refractory Boride.

[140] Ignatiev N.K. (1964). On a practical method for finding dense packings of n-dimensional spheres. Sib. Mat. Zhurnal.

[141] Doraiswamy N., Marks L.D. (1995). Preferred structures in small particles. Philos. Mag. B.

[142] Gammons C.H. (1996). Hydrothermal synthesis of gold grains with apparent five-fold symmetry. Can. Mineral..

[143] Uyeda R., Sunagawa I. (1987). Morphology of Crystals.

[144] Ohashi W., Spaepen F. (1987). Stable Ga–Mg–Zn quasiperiodic crystals with pentagonal dodecahedral solidification morphology. Nature.

[145] Dubost B., Lang J.M., Tanaka M., Sainfort P., Audier M. (1986). Large AlCuLi single quasicrystals with triacontahedral solidification morphology. Nature.

[146] Lundström T. (1997). Structure and Bulk Modulus of High-Strength Boron Compounds. J. Solid State Chem..

[147] Vast N., Baroni S., Zerah G., Besson J.M., Polian A., Grimsditch M., Chervin J.C. (1997). Lattice Dynamics of Icosahedral α-Boron under Pressure. Phys. Rev. Lett..

[148] Kobayashi M., Higashi I., Brodhag C., Thévenot F. (1993). Structure of B_6_O boron-suboxide by Rietveld refinement. J. Mater. Sci..

[149] Lundström T., Andreev Y.G. (1996). Superhard boron-rich borides and studies of the B–C–N system. Mater. Sci. Eng. A.

[150] Bolmgren H., Lundström T., Okada S., Emin D., Aselage T., Beckel C.L., Howard I.A., Wood C. (1991). Boron-Rich Solids.

[151] Donohue J. (1974). The Structures of the Elements.

[152] Hoard J.L., Sullenger D.B., Kennard C.H.L., Hughes R.E. (1970). The structure analysis of β-rhombohedral boron. J. Solid State Chem..

